# The Contrasting Effects of *Bothrops lanceolatus* and *Bothrops atrox* Venom on Procoagulant Activity and Thrombus Stability under Blood Flow Conditions

**DOI:** 10.3390/toxins16090400

**Published:** 2024-09-18

**Authors:** Fatima Radouani, Prisca Jalta, Caroline Rapon, Chloe Lezin, Chelsea Branford, Jonathan Florentin, Jose Maria Gutierrez, Dabor Resiere, Remi Neviere, Olivier Pierre-Louis

**Affiliations:** 1 Cardiovascular Research Team (UR5_3 PC2E), University of the French West Indies (Université des Antilles), 97200 Fort-de-France, France; fatima.radouanisaint-aime@chu-martinique.fr (F.R.); prisca.jalta@chu-martinique.fr (P.J.); caroline.rapon@chu-martinique.fr (C.R.); chloe.lezin@univ-antilles.fr (C.L.); jonathan.florentin@chu-martinique.fr (J.F.); dabor.resiere@chu-martinique.fr (D.R.); ufrsteua@gmail.com (O.P.-L.); 2 Department of Research, University Hospital of Martinique (CHU Martinique), 97261 Fort-de-France, France; 3 Department of Biology, Faculté des Sciences Exactes et Naturelles (SEN), Campus Fouillole, 97157 Pointe-à-Pitre, France; 4 Clodomiro Picado Institute, School of Microbiology, University of Costa Rica, San José 11501, Costa Rica; 5 Department of Toxicology and Critical Care Medicine, University Hospital of Martinique (CHU Martinique), 97261 Fort-de-France, France; 6 Department of Clinical Physiology, University Hospital of Martinique (CHU Martinique), 97261 Fort-de-France, France

**Keywords:** *Bothrops* snake envenoming, *B. lanceolatus*, *B. atrox*, thrombosis, procoagulant, thrombin, kallikrein, shear stress, antivenom

## Abstract

Background: Consumption coagulopathy and hemorrhagic syndrome are the typical features of *Bothrops* sp. snake envenoming. In contrast, *B. lanceolatus* envenoming can induce thrombotic complications. Our aim was to test whether crude *B. lanceolatus* and *B. atrox* venoms would display procoagulant activity and induce thrombus formation under flow conditions. Methods and Principal Findings: Fibrin formation in human plasma was observed for *B. lanceolatus* venom at 250–1000 ng/mL concentrations, which also induced clot formation in purified human fibrinogen, indicating thrombin-like activity. The degradation of fibrinogen confirmed the fibrinogenolytic activity of *B. lanceolatus* venom. *B. lanceolatus* venom displayed consistent thrombin-like and kallikrein-like activity increases in plasma conditions. The well-known procoagulant *B. atrox* venom activated plasmatic coagulation factors in vitro and induced firm thrombus formation under high shear rate conditions. In contrast, *B. lanceolatus* venom induced the formation of fragile thrombi that could not resist shear stress. Conclusions: Our results suggest that crude *B. lanceolatus* venom displays amidolytic activity and can activate the coagulation cascade, leading to prothrombin activation. *B. lanceolatus* venom induces the formation of an unstable thrombus under flow conditions, which can be prevented by the specific monovalent antivenom Bothrofav^®^.

## 1. Introduction

Viperid snakes of the genus *Bothrops* sp. are responsible for the vast majority of snakebites in Central and South America [1,2]. Envenoming by *Bothrops* sp. snakes, such as *B. atrox*, is associated with prominent local injuries, such as myonecrosis, hemorrhage, edema, dermonecrosis, and blistering, as well as systemic effects including coagulopathy, hemorrhage, cardiovascular shock, and acute renal dysfunction [1,2]. Victims of *Bothrops* sp. snakebites often present with major hemostasis abnormalities, such as thrombocytopenia, platelet hypoaggregation, defibrin(ogen)ation, and activation of the clotting pathway by procoagulant bothropic toxins, resulting in clotting factor consumption and coagulopathy [1,2,3].

*B. atrox* is widely distributed in the Amazon regions of many countries. In French Guiana, an overseas territorial collectivity of France, situated on the northeastern coast of South America, In French Guiana, an overseas territorial collectivity of France, situated on the northeastern coast of South America, *B. atrox*, *B. oligobalus*, *B. bilineatus*, and *Lachesis muta* are the viperidae responsible for the most frequent life-threatening envenoming incidents. The annual incidence of *Bothrops* envenoming exceeded 25–35 per 100,000 inhabitants, mainly due to *B. atrox* [4]. Mortality is estimated to be around 0.3/year per 100,000 people [4]. The number of cases may be largely underestimated, due to non-exhaustive reports related to limited epidemiologic center facilities, a large population with irregular migratory status, and people living in the Amazonian Forest, some of whom may have been bitten and died from snakebites without medical assistance. In contrast, *B. lanceolatus* has a much narrower distribution. *B. lanceolatus* is the only venomous snake of the French Lesser Antilles, which is annually responsible for 10–15 envenoming cases/year per 100,000 inhabitants in Martinique [5]. *B. lanceolatus* envenoming is characterized by frequent thrombotic complications rather than systemic bleeding and blood incoagulability [5,6,7,8]. Before the era of antivenom, *B. lanceolatus* envenoming was associated with cerebral, myocardial, or pulmonary infarctions in 25–30% of cases and was fatal in about 10% of cases [6,7,8]. The pathophysiology of thrombosis complicating *B. lanceolatus* envenoming remains poorly understood. Proposed mechanisms of arterial thrombosis in *B. lanceolatus* envenoming include platelet aggregation, a hypercoagulable state, platelet dysfunction, and pro-inflammatory activity, causing endothelial injury, immune-mediated vasculitis, and activation of the innate immune response [3,9]. In addition, while *B. lanceolatus* venom displays a similar proteomic profile compared with those of other *Bothrops* sp. snakes, subtle differences in the peptide sequences of metalloproteinases or serine proteinases may be related to the contrasting biological effects [10,11,12].

Previous studies have yielded conflicting results regarding the procoagulant effects of *B. lanceolatus* venom [10,11,13,14,15,16,17]. Some studies concluded that *B. lanceolatus* venom elicited no procoagulant or defibrinogenating activities, as suggested by the absence of clot formation in citrated human plasma [10,13,14]. These initial findings were unexpected since *B. lanceolatus* envenoming is typically associated with thrombotic complications. Of note, these studies examined the coagulation cascade in citrated plasma without adding calcium and phospholipids, which are crucial co-factors conditioning the level of procoagulant activity of *Bothrops* venom enzymes. However, evidence for a procoagulant effect of *B. lanceolatus* venom was indirectly suggested by the observation that the monospecific *B. lanceolatus* antivenom Bothrofav^®^ could neutralize the procoagulant effects of venoms of other *Bothrops* species. This indicates that horses immunized with *B. lanceolatus* venom have generated antibodies capable of neutralizing some procoagulant toxins of other *Bothrops* sp. [17]. More recently, thrombo-elastography experiments using human plasma [15] or whole blood [11] have suggested that *B. lanceolatus* venom can actually display procoagulant activity in the presence of adequate concentrations of calcium and phospholipids.

Because the thrombotic complications seen in *B. lanceolatus* envenoming are localized on the arterial side of the vasculature [5,6,7,8], mechanisms leading to thrombus formation should be evaluated under high shear rate and shear stress conditions mimicking arterial blood flow [18,19]. A better understanding of the action of *B. lanceolatus* venom on the coagulation cascade will provide further insights for the development of new therapeutic strategies improving the overall care of patients with *B. lanceolatus* envenoming [20,21,22]. While the contribution of specific metalloproteases and serine proteases (and their inhibition) might be considered, the effects of crude venom from wild-caught snakes on coagulotoxicity, both in vitro and under flow conditions [11,15,23], is the first step for the improvement of our understanding of the complex nature of *B. lanceolatus* envenoming.

Therefore, the major objective of our study was to test whether *B. lanceolatus* venom can induce procoagulant activity and thrombus formation under flow conditions, in comparison with the well-known procoagulant *B. atrox* venom. Both *B. lanceolatus* and *B. atrox* venoms from wild-caught snakes will be studied. The specific objectives were to assess (i) in vitro procoagulant activity; (ii) the hydrolysis of chromogenic substrates used to determine the activity of enzymes in the coagulation cascade; (iii) fibrinolytic activities on human fibrinogen; and (iv) thrombus formation under high shear rate and shear stress conditions, using crude venom obtained from adult wild *B. lanceolatus* and *B. atrox* specimens. In addition, the effectiveness of the monovalent antivenom Bothrofav^®^ on the coagulant toxicity of *B. lanceolatus* venom was determined.

## 2. Results

### 2.1. Evaluation of the In Vitro Procoagulant Effect of B. lanceolatus Venom

**Figure 1:**
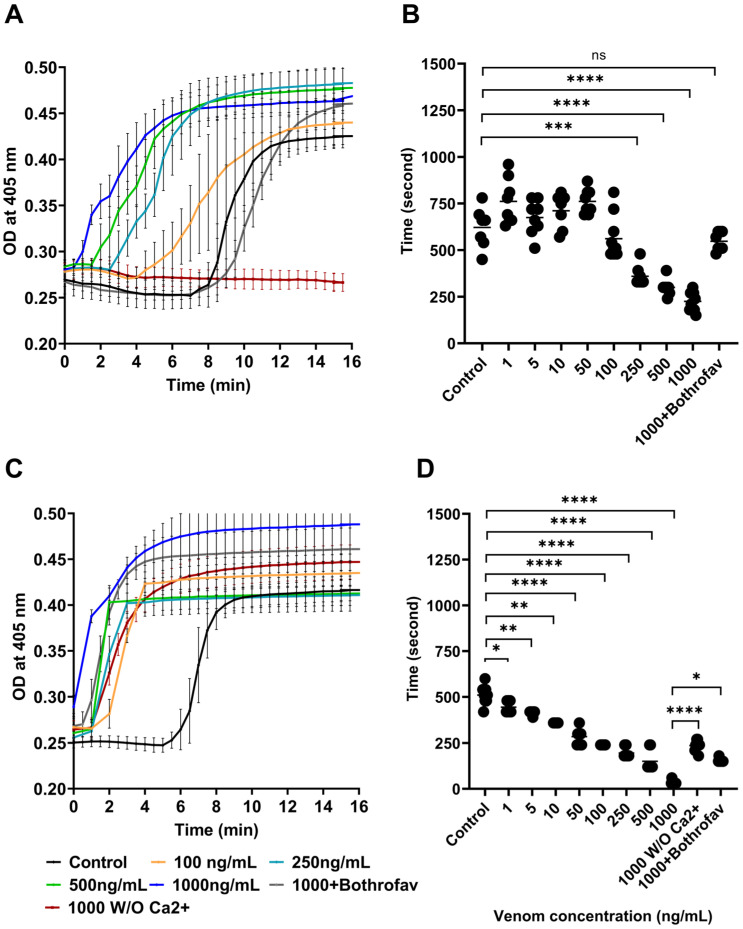
Effect of *B. lanceolatus* and *B. atrox* on fibrin formation, measured as optical density (OD) increased at 405 nm. (**A**) Kinetics of fibrin formation in the presence of *B. lanceolatus* venom. (**B**) Time of recalcification in the presence of *B. lanceolatus* venom. (**C**) Kinetics of fibrin formation in the presence of *B. atrox* venom. (**D**) Time of recalcification in the presence of *B. atrox* venom. Control 0 ng/mL (black curve), 100 ng/mL (yellow curve), 250 ng/mL (blue curve), 500 ng/mL (green curve), 1000 ng/mL (dark blue curve), and 1000 ng/mL without calcium (brown curve). Bothrofav^®^ prevented the procoagulant activity of crude *B. lanceolatus* venom at a venom concentration of 1000 ng/mL (grey curve). Data are mean ± SD; *n* = 6–8; ns: non-significant; *, **, *** and **** indicate *p* < 0.05, *p* < 0.01, *p* < 0.001 and *p* < 0.0001 compared with controls, respectively.

Firstly, we ascertained that *B. lanceolatus* venom was able to generate fibrin formation in human plasma in vitro under static conditions (Figure 1A). The generation of fibrin began earlier for concentrations of *B. lanceolatus* venom ranging from 250 ng/mL (191 ± 22 s; *p* < 0.0001) to 1000 ng/mL (53 ± 21 s; *p* < 0.0001), in comparison with controls (438 ± 86 s). Under a concentration of 100 ng/mL, *B. lanceolatus* venom had no effect on fibrin formation (Figure 1A). These results suggest a procoagulant effect of *B. lanceolatus* venom in comparison with spontaneous coagulation in human recalcified plasma. Importantly, *B. lanceolatus* venom was unable to initiate coagulation in the absence of Ca^2+^ (shown as “1000 W/O Ca^2+^” in Figure 1A). Next, the time of maximal fibrin formation was evaluated (Figure 1B). In comparison to spontaneous coagulation time (621 ± 109 s), concentrations of *B. lanceolatus* venom > 100 ng/mL decreased the time of maximal fibrin formation. A concentration of 250 ng/mL of *B. lanceolatus* venom induced a significant decrease in the time of maximal fibrin formation (360 ± 53 s; *p* < 0.0001), which was further reduced with concentrations of *B. lanceolatus* venom of 1000 ng/mL (225 ± 51 s; *p* < 0.0001) (Figure 1B). In comparison with the control and *B. lanceolatus* venom conditions, the generation of fibrin in human plasma began earlier in *B. atrox* venom conditions, even in the absence of Ca^2+^ (Figure 1C). The time to achieve maximum fibrin formation was gradually reduced as *B. atrox* venom concentrations increased (Figure 1D). The maximum level of fibrin generation occurred significantly earlier with almost 1 ng/mL (445.7 ± 32.07 s; *p* < 0.05), compared to the control at *p* < 0.05, and took only 33.75 ± 10.61 s with 1000 ng/mL of *B. atrox* venom.

**Figure A1:**
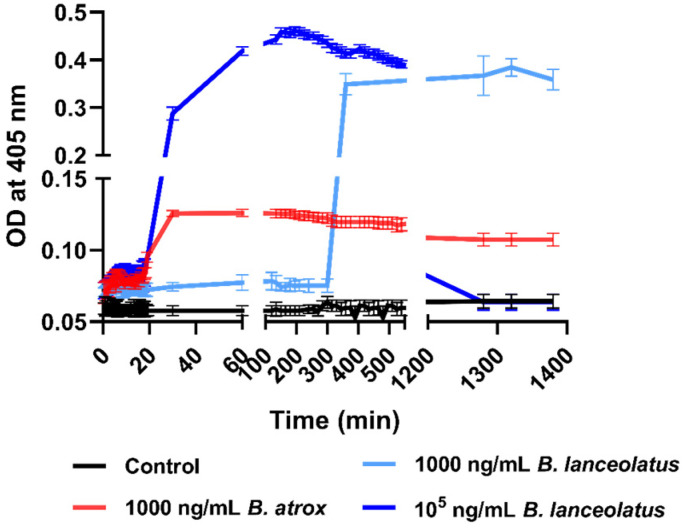
Fibrinogen clotting time. Evolution of fibrinogen clotting time, determined as the evolution of optical density at 405 nm over time in the presence of 1000 ng/mL of *B. atrox* (red curve), 1000 ng/mL of *B. lanceolatus* at 1000 ng/mL (blue), 10^5^ ng/mL (dark blue), and the control (black curve).

In order to document the thrombin-like activity of *B. lanceolatus* venom, we tested its clotting activity with human fibrinogen (Figure A1). A concentration of 10^5^ ng/mL of *B. lanceolatus* venom induced clot formation within 60 min (time to obtain maximal OD), while a concentration of 1000 ng/mL induced clot formation within 10 h (time to obtain maximal OD) (Figure A1).

Overall, the results displayed in Figure 1 underscore the in vitro coagulant activity of *B. lanceolatus* and *B. atrox* venoms in the presence of calcium under static conditions. Bothrofav^®^ fully prevented the procoagulant activity of crude *B. lanceolatus* venom at a venom concentration of 1000 ng/mL and also prevented, at least in part, the procoagulant effect of *B. atrox* venom at the same concentration.

### 2.2. Fibrinogenolytic Activity

**Figure 2:**
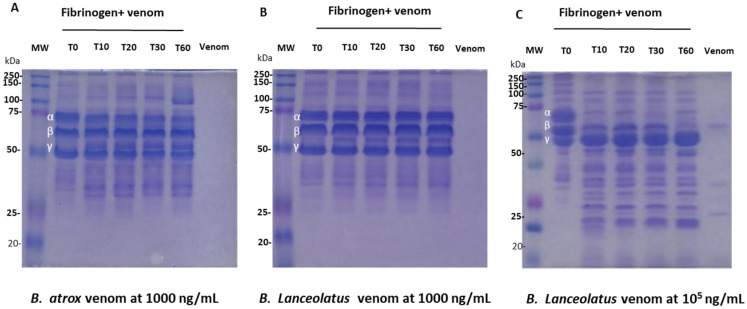
Effect of *B. atrox* and *B. lanceolatus* venoms upon purified human fibrinogen. (**A**) Fibrinogenolytic activity of *B. atrox* venom at 1000 ng/mL. (**B**) Fibrinogenolytic activity of *B. lanceolatus* venom at 1000 ng/mL and (**C**) at 10^5^ ng/mL, assessed by reducing SDS-gel electrophoresis (12%). Column 1: molecular mass standard; column 2: fibrinogen control; column 3: fibrinogen incubated with venom for 10 min; column 4: fibrinogen incubated with venom for 20 min; column 5: fibrinogen incubated with venom for 30 min; column 6: fibrinogen incubated with venom for 60 min; column 7: 1000 ng/mL of *B. atrox* and *B. lanceolatus* venom for (**A**) and (**B**), respectively, and 10^5^ ng/mL of *B. lanceolatus* venom alone.

The fibrinogenolytic activity of *B. atrox* and *B. lanceolatus* venoms was determined using SDS-PAGE (Figure 2). In the presence of *B. atrox* venom at 1000 ng/mL concentration (Figure 2A), fibrinogen degradation over time was noted (Figure 2A). From the first minutes of incubation, the Aα chain was strongly degraded (lines 3 to 6), and a slight degradation of the γ chain (lines 5 and 6) was observed after 30 min of incubation (Figure 2A). The Bβ chain did not show any degradation (Figure 2A). In the presence of *B. lanceolatus* venom at 1000 ng/mL concentration, a degradation product with a molecular weight of 60 kDa was observed after 10 min of incubation (Figure 2B, columns 3–6). Compared with the control (Figure 2B, column 2), *B. lanceolatus* venom at 1000 ng/mL concentration induced only limited degradation of Aα and/or Bβ chains (Figure 2B, columns 3–6). When fibrinogen was incubated with *B. lanceolatus* venom at a concentration of 10^5^ ng/mL (Figure 2C), pronounced degradation of Aα occurred after 10 min of incubation (Figure 2C, column 3), followed by complete degradation of the Bβ chain after 60 min of the reaction (Figure 2C, column 6).

### 2.3. Evaluation of Coagulation Factor Activities of Venoms

Evaluation of the coagulation factor activities was performed in citrated plasma with the presence of a physiological concentration of Ca^2+^ to initiate the activation of coagulation factors and fibrin formation. The activation of coagulation factors elicited by *B. lanceolatus* and *B. atrox* venoms was assessed using synthetic specific substrates coupled to p-nitroaniline (pNAPEP). The synthetic substrates displayed similar selectivity as the natural substrates of a specific enzyme. Synthetic substrates consist of a sequence of amino acids with a chromogen group (pNA) at the terminal. The substrate is cleaved by the enzyme to be determined and releases the chromophore, which can be photometrically measured. In our experimental protocol, increases in pNA release may be due to either the enzymatic activity of the venom (i.e., *B. lanceolatus* or *B. atrox* venoms without plasma) or the venom-induced activation of plasmatic coagulation factors (i.e., *B. lanceolatus* or *B. atrox* venoms with plasma). Whether pNA release is higher in plasma than in crude venom (without plasma) indicates that the venom has actually activated the coagulation factors, in addition to its enzymatic activity. 

**Figure 3:**
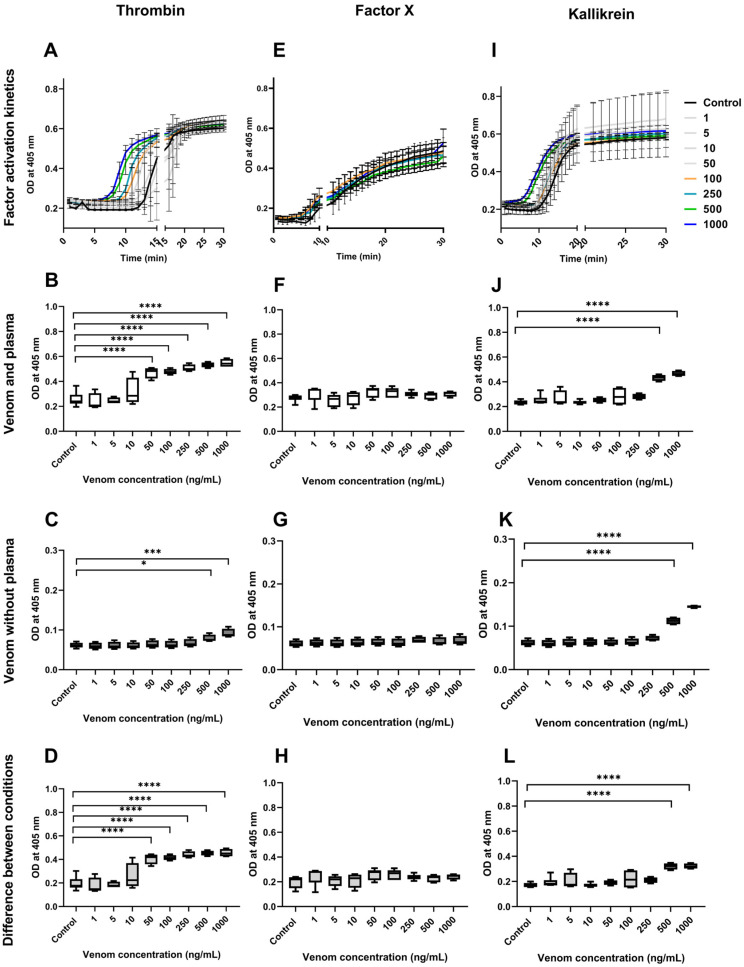
Activation of coagulation factors by *B. lanceolatus* venom. Kinetics of the activation of prothrombin (**A**); releases of pNA (0216) in plasmatic conditions (**B**), non-plasmatic conditions (**C**) and differences in OD between the two conditions (**D**) in the presence of various concentrations of *B. lanceolatus* venom; (**E**) kinetics of the activation of Factor X; releases of pNA (1032) in plasmatic conditions (**F**); non-plasmatic conditions (**G**) and differences in OD between the two conditions (**H**) in the presence of various concentrations of *B. lanceolatus* venom; (**I**) kinetics of the activation of prekallikrein; release of pNA (1266) in plasmatic conditions (**J**); non-plasmatic conditions (**K**) and differences in OD between the two conditions (**L**) in the presence of various concentrations of *B. lanceolatus* venom. The release of pNa in plasmatic conditions is due to either the activation of coagulation factor and/or the amidolytic activity of venom enzymes. Direct cleavage of pNAPEP 0216 (thrombin), pNAPEP 1032 (FXa), and pNAPEP 1266 (kallikrein) by crude venom (non-plasmatic conditions) indicates the increased amidolytic activity of the venom on the studied substrates. Positive differences in OD between plasmatic and non-plasmatic conditions indicate the activation of plasmatic clotting factors, i.e., prothrombin to thrombin and prekallikrein to kallikrein, respectively. Data are mean ± SD; *n* = 4–8; *, *** and **** indicate *p* < 0.05, *p* < 0.001 and *p* < 0.0001, compared with the controls, respectively.

**Figure 4:**
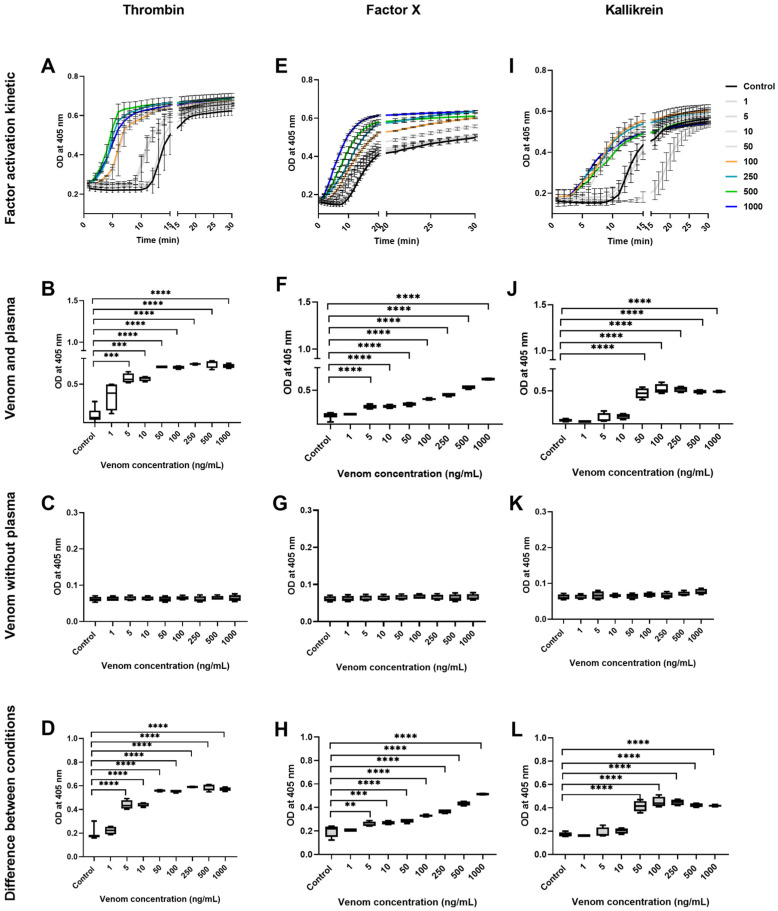
Activation of coagulation factor by *B. atrox* venom. (**A**) Kinetics of the activation of prothrombin; releases of pNA (0216) in plasmatic conditions (**B**); non-plasmatic conditions (**C**) and differences in OD between the two conditions (**D**) in the presence of various concentrations of *B. atrox* venom. (**E**) Kinetics of the activation of Factor X; releases of pNA (1032) in plasmatic conditions (**F**); non-plasmatic conditions (**G**) and differences in OD between the two conditions (**H**) in the presence of various concentrations of *B. atrox* venom. (**I**) Kinetics of the activation of prekallikrein; releases of pNA (1266) in plasmatic conditions (**J**); non-plasmatic conditions (**K**) and differences in OD between the two conditions (**L**) in the presence of various concentrations of *B. atrox* venom. Release of pNa in plasmatic conditions is due to either the activation of a coagulation factor and/or the amidolytic activity of venom enzymes. The direct cleavage of pNAPEP 0216 (thrombin), pNAPEP 1032 (FXa), and pNAPEP 1266 (kallikrein) by crude venom (non-plasmatic conditions) indicates the increased amidolytic activity of the venom on the studied substrates. Positive differences in optical density between plasmatic and non-plasmatic conditions indicate the activation of plasmatic clotting factors, i.e., prothrombin to thrombin and prekallikrein to kallikrein, respectively. Data are mean ± SD; *n* = 4–8; **, *** and **** indicate *p* < 0.001, *p* < 0.001 and *p* < 0.0001 compared with the controls, respectively.

To assess whether *B. lanceolatus* and *B. atrox* venoms induce the earlier activation of coagulation factors compared with controls, the kinetics of prothrombin, FX, and kallikrein activation were followed for 30 min in the absence or in the presence of various concentrations of venoms. Our results show the earlier activation of prothrombin and kallikrein, but not of FX, in comparison to the control in *B. lanceolatus* venom conditions (Figure 3A,E,I). In addition, the earlier activation of prothrombin, FX, and kallikrein was observed in *B. atrox* venom conditions (Figure 4A,E,I) in a concentration-dependent manner during the first 10 min of the reaction. However, after 10 min, the differences in comparison to the control disappeared, to become undetectable after 15 min of incubation in all conditions. Thus, statistical comparisons between venom conditions were carried out at 10 min.

In crude venom conditions (without plasma), *B. lanceolatus* venom showed a significant enhancement of amidolytic activities on synthetic substrates (thrombin and kallikrein) for the two highest concentrations of the venom (Figure 3C,G,K). In contrast, *B. atrox* venom showed no significant increase in amidolytic activity (Figure 4C,G,K). In order to evaluate whether *B. lanceolatus* venom can activate coagulation factors independently of its enzymatic activity, the OD differences between conditions with plasma (Figure 3B,F,J) and without plasma (Figure 3D,H,L) were calculated. Compared with controls, *B. lanceolatus* elicited a significant increase in thrombin (*p* < 0.0002) with a concentration of venom equal to or greater than 50 ng/mL (Figure 3D) and with kallikrein (*p* < 0.002) from 500 ng/mL (Figure 3L) of venom concentration. 

In contrast, *B. atrox* venom activated the coagulation factors but did not cleave synthetic substrates, as the OD differences (Figure 4D,H,L) between conditions with plasma (Figure 4B,F,J) and without plasma (Figure 4C,G,K) were increased. These results suggest that *B. atrox* venom elicits the formation of activated coagulation factors when incubated with plasma. 

Comparison of the respective ability of *B. lanceolatus* and *B. atrox* venoms to induce synthetic pNAPEP (pNAPEP 0216 (thrombin), pNAPEP 1032 (FXa), and pNAPEP 1266 (kallikrein) cleavage are summarized in Figure A3. At a concentration of 1000 ng/mL, the crude *B. lanceolatus* venom displayed consistent increased thrombin-like and kallikrein-like activities (0.10 ± 0.01 and 0.15 ± 0.01 optical density at 405 nm, respectively), while the cleavage of pNAPEP 1032 (FXa) was weaker (0.07 ± 0.01 optical density at 405 nm) (Figure A3). In comparison with crude *B. lanceolatus* venom, crude *B. atrox* venom displayed weaker amidolytic activity with the studied pNAPEPs (Figure A4).

**Figure 5:**
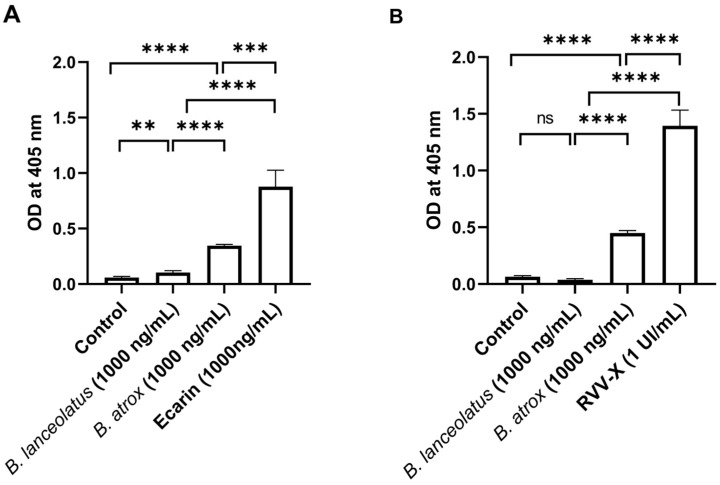
Assessment of the activation of purified human coating factors. (**A**) Activation of purified human prothrombin. (**B**) Activation of purified human FX in the presence of *B. lanceolatus* and *B. atrox* venoms at 1000 ng/mL. The thrombin-activator venom protease ecarin and coagulation FX activating enzyme from Russell’s viper venom (RVV-X) were used as positive controls at 1000 ng/mL and 1 UI/mL, respectively. Data are mean ± SD; *n* = 4–6; ns: non-significant; **, *** and **** indicate *p* < 0.01, *p* < 0.001 and *p* < 0.0001 compared with controls, respectively.

In addition to the assessment of plasmatic clotting factor activation, the activation of purified human prothrombin and FX in the presence of *B. lanceolatus* and *B. atrox* venoms at 1000 ng/mL was studied. For this purpose, 1000 ng/mL of the prothrombin-activator venom protease ecarin and 1 UI of the coagulation FX-activating enzyme from Russell’s viper venom (RVV-X) were used as the positive controls. The results show the consistent activation of prothrombin and FX with ecarin and RVV-X, respectively. Whereas *B. atrox* also directly activated purified human prothrombin and FX, *B. lanceolatus* venom had a weaker activity with human prothrombin but no activity with purified human FX (Figure 5A,B).

### 2.4. Thrombus Formation Analysis in Flow Conditions

**Figure 6:**
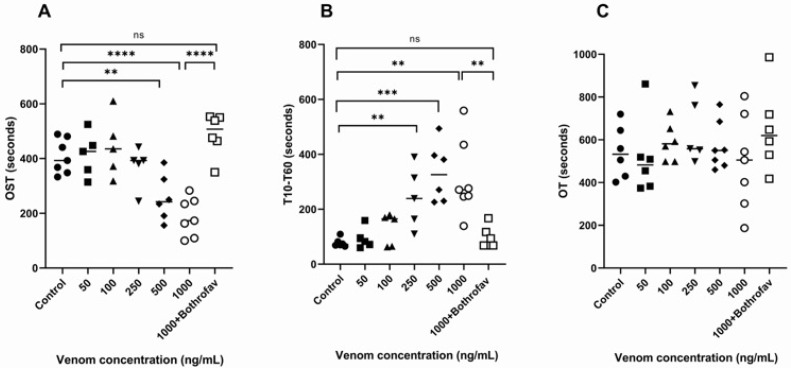
Whole blood coagulation activity (AR-Chip) in the presence of *B. lanceolatus* venom. (**A**) Thrombus formation displayed as the changes in occlusion starting time (OST, s); (**B**) thrombus formation growth rates (T_10_–T_60_ time, s); and (**C**) occlusion time (OT, s). Data are mean ± SD; *n* = 5–7; ns: non-significant, **, *** and **** indicate *p* < 0.01, *p* < 0.001, and *p* < 0.0001, compared with controls, respectively.

The whole blood coagulation activity of *B. lanceolatus* and *B. atrox* venoms under flow and high shear rate conditions was assessed using a T-TAS device. In the AR-Chip, platelets are activated by the effect of collagen and 600/s wall shear stress, and the blood coagulation pathway is activated by tissue thromboplastin and calcium ions. Thrombi are formed as a result of primary and secondary (especially with extrinsic pathway) hemostasis, and the pressure inside the flow path increases due to the occlusion of the flow paths. Figure 6 displays the significant decreases in OST (Figure 6A) in the presence of *B. lanceolatus* venom for concentrations of 500 ng/mL (257 ± 35 s; *p* < 0.001) and 1000 ng/mL (187 ± 26 s; *p* < 0.0001), compared with the controls (408 ± 24 s). Bothrofav^®^ fully prevented the reduction of OST that was induced by *B. lanceolatus* venom at a venom concentration of 1000 ng/mL (Figure 6A). In comparison to control conditions (78 ± 16 s), the rates of thrombus formation growth (T_10_–T_60_) were significantly increased with a *B. lanceolatus* venom concentration ranging from 250 ng/mL (243 ± 113 s; *p* = 0.002) to 1000 (310 ± 140 s; *p* = 0.005) (Figure 6B). Occlusion time (OT) in the control and *B. lanceolatus* venom conditions was similar since OST was earlier and T_10_–T_60_ was longer (Figure 6C).

**Figure A2:**
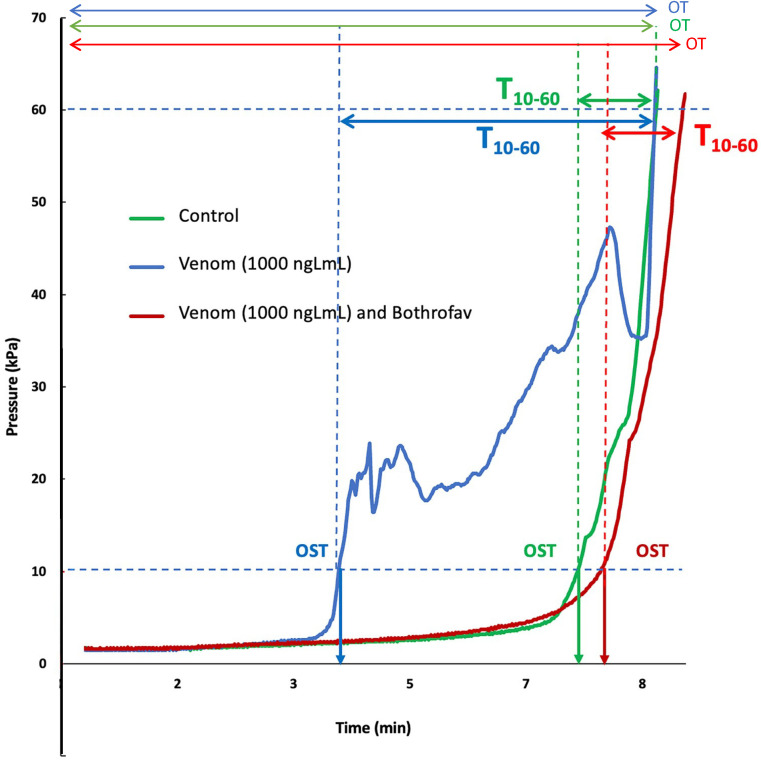
Traces displaying thrombus formation. The occlusion start time (OST) for the control, *B. lanceolatus* (with and without Bothrofav^®^), at 1000 ng/mL is indicated. OST refers to the lag time for the flow pressure to reach 10 kPa, due to partial occlusion of the capillary. Occlusion time (OT) represents the lag time for the flow pressure to reach 60 kPa from the baseline pressure. Thrombus formation growth rates (T_10_–T_60_) refer to the lag time for the flow pressure to reach 60 kPa from 10 kPa of pressure.

**Figure A3:**
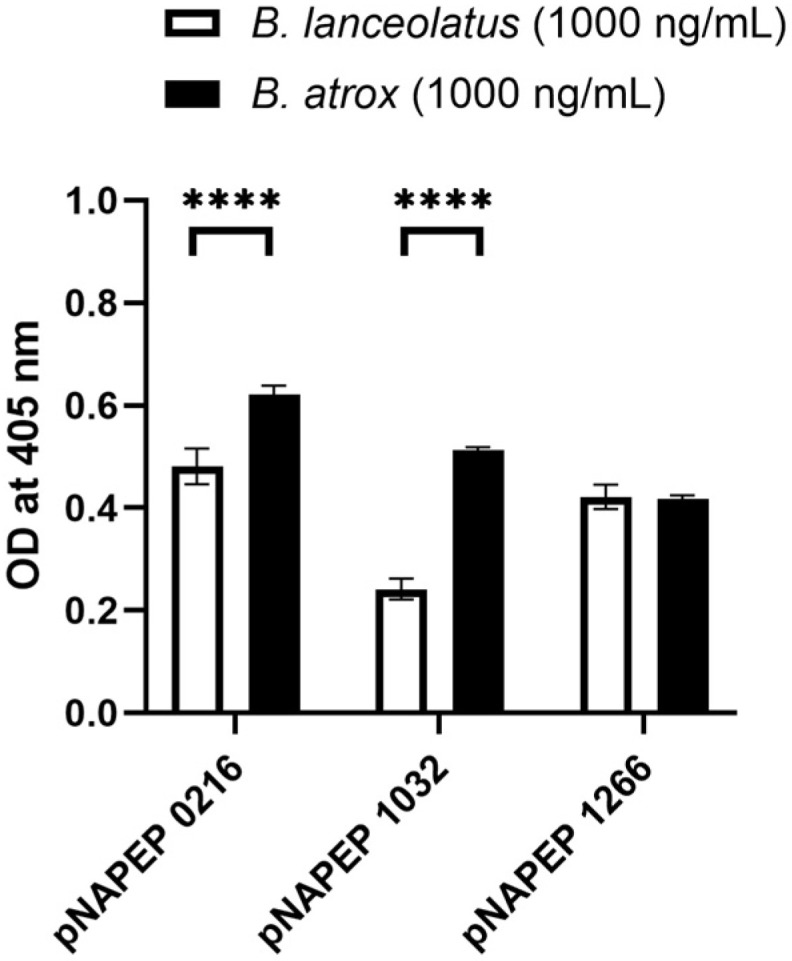
Comparison of the respective abilities of *B. lanceolatus* and *B. atrox* venoms to activate coagulation factors at 1000 ng/mL, using the synthetic peptides, pNAPEP 0216, 1032, and 1266, which are the specific synthetic chromogenic substrates for the measurement of thrombin, FXa, and kallikrein activities. Results are expressed as increases in optical density (OD) at 405 nm. Data are the mean ± SD; *n* = 4–6; **** indicates *p* < 0.0001.

**Figure A4:**
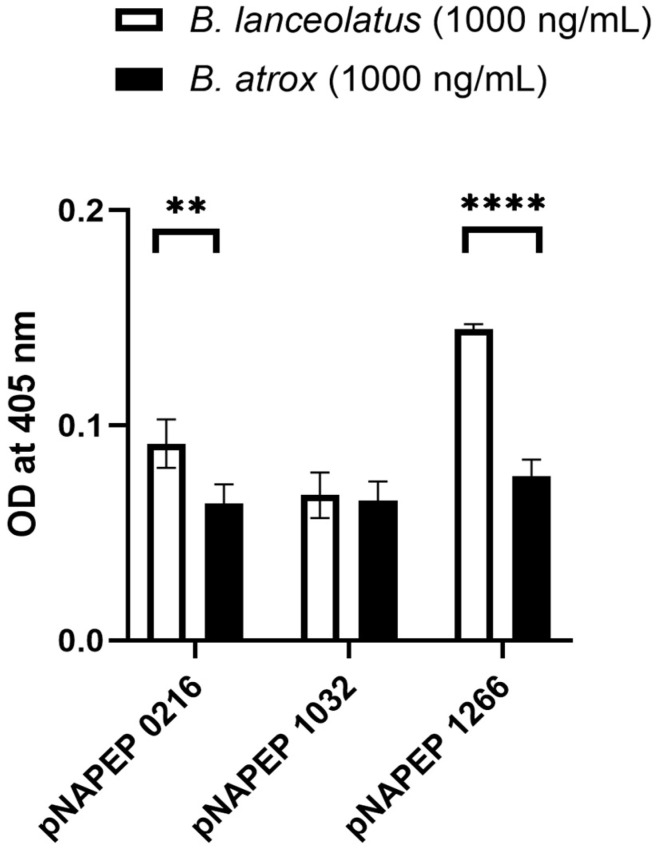
Comparisons of the respective amidolytic activity of crude *B. lanceolatus* and *B. atrox* venoms at 1000 ng/mL on the synthetic peptides, pNAPEP 0216, 1032, and 1266, which are the specific synthetic chromogenic substrate for the measurement of thrombin, FXa, and kallikrein activities. The results are expressed as increases in optical density (OD) at 405 nm. Data are mean ± SD; *n* = 4–6; ** and **** indicate *p* < 0.02 and *p* < 0.0001, respectively.

The pressure traces (T_10_–T_60_) displayed in *B. lanceolatus* venom experiments showed a pattern of periodic drops in pressure, suggesting the fragility of formed thrombi that could not resist the shear stress (Figure A2). Bothrofav^®^ fully prevented the increase in T_10_–T_60_ induced by *B. lanceolatus* venom at a venom concentration of 1000 ng/mL (97 ± 40 s) (Figure 6B). Bothrofav^®^ also prevented the abnormal profile observed on T_10_–T_60_ pressure traces induced by *B. lanceolatus* venom at a venom concentration of 1000 ng/mL (Figure A2).

**Figure 7:**
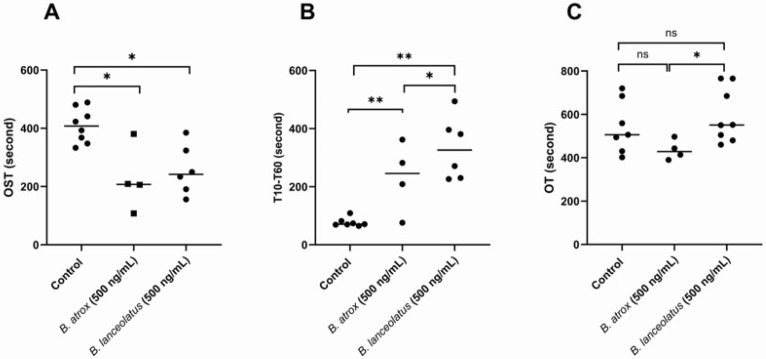
Comparison of whole blood coagulation activity (AR-Chip) of *B. atrox* and *B. lanceolatus* venoms. (**A**) Comparisons of occlusion starting times (OST, s), (**B**) T_10_-T_60_ time (s) and (**C**) occlusion time (OT, s) in whole blood coagulation activity (AR-Chip) in the presence of *B. lanceolatus* and *B. atrox* venoms at 500 ng/mL. Data are mean ± SD; *n* = 4–8; ns: non-significant; * and ** indicate *p* < 0.01 and *p* < 0.05, respectively.

Next, comparisons between *B. lanceolatus* and *B. atrox* venom experiments were performed using 500 ng/mL and 1000 ng/mL venom concentrations. At a concentration of 500 ng/mL, *B. atrox* venom induced a similar reduction in OST (Figure 7A), but the lag time to reach the maximal pressure of 60 kPa from 10 kPa (T_10_–T_60_) increased less markedly (Figure 7B) compared to that under *B. lanceolatus* venom conditions. Occlusion time under *B. atrox* venom conditions tended to be lower compared with *B. lanceolatus* venom condition (Figure 7C), in the direction of reduced coagulation time. Compared with *B. lanceolatus* venom, *B. atrox* venom at a concentration of 1000 ng/mL induced immediate clotting in the AR-Chip, suggesting the formation of firm thrombi.

Table A1 summarizes the conclusion of each experiment performed regarding the procoagulant effects, clotting factor activation, and amidolytic activities.

## 3. Discussion

Despite similarities in some aspects of the clinical presentation described after *Bothrops* sp. snakebite envenoming, *B. lanceolatus* snakebites are not associated with classical bothropic systemic bleeding and consumption coagulopathy. Instead, untreated *B. lanceolatus* envenoming may be complicated by multiple systemic arterial thrombosis within 48 h of the bite when antivenom administration is delayed or absent [5,6,7,8]. Surprisingly, the thrombotic manifestations of *B. lanceolatus* envenoming were observed in the absence of in vitro procoagulant effects or in vivo defibrinogenating activities, as suggested by the absence of clot formation in citrated human plasma and prothrombin or factor X activation [10,13,14]. Indeed, *B. lanceolatus* venom dose-dependently clotted purified human fibrinogen, indicating the presence of a thrombin-like enzyme [9], but was devoid of defibrinating activity after venom injection in mice [10,13,14,24]. The absence of any in vitro procoagulant activity of *B. lanceolatus* venom has recently been challenged [15,16,17] and the inconsistency of the results has been attributed to differences in experimental methodology, including venom concentrations, the addition of cofactors such as calcium or phospholipids, and the types of blood-derived products used [3,11].

The results of the current work underscore the in vitro procoagulant activity of *B. lanceolatus* venom in the presence of adequate cofactors. In static conditions, *B. lanceolatus* venom induced fibrin generation in human recalcified plasma. The lowest venom concentration that was able to reduce the time to obtain maximal fibrin formation was much lower in *B. atrox* venom (1–5 ng/mL) compared with *B. lanceolatus* venom (100–250 ng/mL), which suggests a weaker procoagulant effect of the *B. lanceolatus* venom. Of note, the monospecific antivenom Bothrofav^®^ fully prevented the in vitro procoagulant effects of *B. lanceolatus* venom. Our results also suggest that *B. lanceolatus* venom functions in a fashion similar to snake venom serine proteases, which exhibit fibrin(ogen)olytic activity. This contention is supported by an analysis of the time course of human fibrinogen cleavage, showing that the proteases present in *B. lanceolatus* venom were able to induce mild degradation of Aα and/or Bβ chains. Of note, fibrinogen cleavage induced by *B. lanceolatus* venom was found to be less pronounced compared with *B. atrox* venom, which induced the fast and complete degradation of the Aα chain and slight degradation of the γ chain. Overall, we concluded that *B. lanceolatus* venom displays procoagulant capabilities when tested on human plasma supplemented with calcium and phospholipids, which was related to the prothrombin activator(s) but no factor X activator(s).

Incubation of the venom with plasma confirmed that *B. lanceolatus* venom was able to induce thrombin-like and kallikrein-like activities, but no FXa activity. In contrast, *B. atrox* venom displayed thrombin-like and kallikrein-like, as well as FXa-like activities, confirming the well-known potent procoagulant effects of this venom. The absence of the ability of *B. lanceolatus* venom to activate FX was confirmed using purified human FX proteins. As previously shown [10,13,14], the incubation of *B. lanceolatus* venom with purified FX did not lead to factor X activation. In comparison, enzymes in *B. atrox* venom were able to activate purified human factor X in FXa, which then cleaved the synthetic substrate added to the buffer. Using the same rationale, increases in plasmatic thrombin-like, FXa-like, and kallikrein-like activities may be related to either the activation of coagulation factor zymogens in the plasma or the enzyme activities of the venoms cleaving the specific chromogenic substrates. In the case of *B. atrox,* increased thrombin-like, FXa-like, and kallikrein-like activities were only related to zymogen cleavage in the plasma, with no detectable enzyme activities by the venom. In contrast, increased plasmatic thrombin-like and kallikrein-like activities induced by *B. lanceolatus* venom were attributed to both the activation of coagulation factor zymogens present in the plasma and the enzyme activities of the venom. Overall, the results of our experiments suggest that *B. lanceolatus* venom has specific effects on blood coagulation, which are different from the well-established procoagulant effects of most *Bothrops* sp. venoms, including *B. atrox* venom. Our results confirm that *B. lanceolatus* venom displays procoagulant activity when adequate cofactors are used. The weak fibrin(ogen)olytic activity of *B. lanceolatus* venom observed in our study is consistent with previous studies showing no defibrinogenating effects in vivo after its intravenous injection in mice and only a minimal reduction in plasmatic fibrinogen concentration in envenomed humans [4,11,24].

Under flow and high shear rate conditions, both *B. lanceolatus* and *B. atrox* venoms were able to initiate clot formation. These venoms reduced the occlusion start time (OST), suggesting that the onset of clotting started earlier when venom was added to the system, compared with the control conditions. As a result of reduced OST, the rate of thrombus formation growth, i.e., lag time for maximal pressure (T_10_–T_60_), was increased in *B. lanceolatus* and *B. atrox* venom conditions. In the case of *B. lanceolatus* venom, these changes were associated with periodic and abrupt drops in pressure patterns in *B. lanceolatus* venom, suggesting the fragility of formed thrombi that could not resist shear stress. In contrast, *B. atrox* venom induced immediate and bulk plugging of the microfluidic channels of the flow chamber system, suggesting firm thrombus formation. In high shear rate conditions, the monospecific antivenom Bothrofav^®^ fully prevented the procoagulant effects of *B. lanceolatus* venom. Interestingly, Bothrofav^®^ also corrected, at least in part, the procoagulant effect of *B. atrox* venom, which is consistent with previous studies showing that this antivenom is able to neutralize the coagulotoxins of *B. atrox* venom [15,17]. Whether the fragility of formed thrombi, producing weak, transient clots that are easily broken down, is responsible for the formation of multiple arterial thrombotic complications in *B. lanceolatus* envenoming cases [5,6,7,8,9,25], or whether other pathophysiological processes are involved deserves further specific studies.

Thrombus fragility has previously been associated with some *Bothrops* sp. venoms displaying so-called pseudo-procoagulant activity, whereby fibrinogen is cleaved by thrombin-like proteases with procoagulant and kallikrein-like type serine protease activity, producing weak, transient, and poorly cross-linked clots that are easily broken down [26,27,28,29,30,31,32]. In our study, crude *B. lanceolatus* venom displayed kallikrein-like activity, while *B. atrox* venom displayed no detectable kallikrein-like activity. While the exact significance of these discrepancies is unclear, previous studies have reported the association between venom, kallikrein-like activity, and acute inflammation in various venomous animal species [33,34]. Hence, we speculate that the elevated kallikrein-like activity of *B. lanceolatus* venom could trigger additional pathways independently of the coagulation cascade [26,27,28,29,30,31,32]. For example, experimental studies have previously reported that compared with *B. atrox* venom, *B. lanceolatus* venom was more prone to inducing endothelial activation and a systemic inflammatory response [19,35,36]. Indeed, kallikrein belongs to a family of serine proteases initially known to generate vasoactive kinins from *α* kininogens, which are involved in the regulation of blood pressure via the renin-angiotensin pathway, vascular permeability, and inflammatory processes [37,38,39,40]. Kallikrein plays a crucial role in the activation of the complement system, and, thus, the immune response. Specifically, kallikrein can cleave the complement components C5 and C3 and can activate the terminal complement pathway [39,40]. In line with this finding, *B. lanceolatus* venom strongly induces the generation of anaphylatoxins, such as C5a and C4a, and the terminal complement complex in human serum [35]. *B. lanceolatus* venom also cleaves the purified human components C3, C4, and C5, with the production of biologically active C5a [35]. Hence, kallikrein-induced inflammation could be involved in the mechanisms leading to thrombotic complications in *B. lanceolatus* envenoming. We speculate that compared with *B. atrox* venom, higher kallikrein-like activity in crude *B. lanceolatus* venom would be more prone to inducing thrombo-inflammation and thrombotic complications in envenomed humans.

Our study has some limitations. Firstly, we mainly used in vitro approaches to characterize the immediate effects of *B. lanceolatus* venom on coagulation and thrombus formation. It is, hence, warranted to develop additional dynamic approaches based on in vivo animal models to study the mechanisms of thrombosis in *B. lanceolatus* envenoming. Of note, previous attempts have failed to reproduce the thrombotic complications observed in *B. lanceolatus*-envenomed humans in rodent models [12]. The use of a total thrombus formation analysis system (T-TAS) or rotational thrombo-elastometry (ROTEM) for assessing hemostatic disturbances in envenomed patients would provide useful information for understanding the coagulopathic effects induced by *B. lanceolatus* venom. Another limitation of this study is that experiments have only focused on coagulation alterations, while *B. lanceolatus* envenoming is a complex condition involving many pathophysiological events, including inflammatory events, which could influence the hemostasis process.

## 4. Conclusions

Overall, this study vastly improves our fundamental knowledge base on the coagulotoxic action of *B. lanceolatus* venom. In plasma conditions, *B. lanceolatus* venom was able to activate prothrombin but not FX. Under flow and high shear rate conditions, *B. lanceolatus* venom increased the rate of thrombus formation, suggesting abnormal coagulation processes and an induced fragility of formed thrombi that could not resist shear stress. Overall, our results confirm that *B. lanceolatus* venom displays thrombin-like activity but is devoid of actual procoagulant activity as defined by the activation of zymogens present in the plasma, leading to coagulation factor activation.

## 5. Materials and Methods

### 5.1. Ethics Statement

The human donors were adult healthy volunteers who did not report the use of any medication affecting hemostasis during the 30 days before blood collection. Subjects signed a written consent document in accordance with the Declaration of Helsinki and national regulations. Samples of human whole blood from healthy donors, collected in 4.5 mL Vacutainer™ tubes containing a solution of 3.2% sodium citrate and the remains after analysis (known as residues), were obtained from the Biological Resource Center of the University Hospital of Martinique. The study was approved by the local Medico-Scientific Committee and Institutional Review Board (IRB 2024#1222) of the University Hospital of Martinique (article L. 1243-3 Code de Santé Publique).

### 5.2. Venom and Antivenom

The crude venom was obtained from adult wild *B. lanceolatus* (*n* = 6) and *B. atrox* (*n* = 4) specimens captured in Martinique and French Guiana, respectively [19]. Venom samples from adult specimens were pooled, lyophilized (Freezone, Labconco, Kansas City, MO, USA), and stored at −80 °C until use (stock solution of 10 mg/mL in water). The monospecific antivenom Bothrofav^®^ (Sanofi Pasteur, Lyon, France) was used (batch J8216; protein concentration of 20.7 ± 0.05 g/dL) in the experiments at a final concentration of 0.21 mg/mL. Bothrofav^®^ is a preparation containing F(ab’)_2_ fragments neutralizing *B. lanceolatus* venom toxins. In the neutralization experiments, the antivenom was incubated with plasma or whole blood prior to the addition of venoms. No effects of antivenom alone were observed.

### 5.3. Plasma and Fresh Blood Samples

Whole fresh human blood was collected from volunteers using 3.2% sodium citrate Vacutainer™ tubes. The human sample size was *n* = 8, which included healthy donors with ages ranging from 28 to 65 years (4 females and 4 males). Lyophilized standard coagulation control plasma was used as advised by the supplier (control N 5020050, Cryopep, Montpellier, France).

### 5.4. Coagulant Activity and Fibrin Formation

The coagulant activity of *B. lanceolatus* and *B. atrox* venoms was measured, following a modified protocol described elsewhere. Briefly, a solution containing 53 µL of HEPES-saline buffer (10 mM HEPES, 150 mM NaCl, pH = 7.4), supplemented or not supplemented with 10 µL of CaCl_2_ (2.5 mM final concentration), was warmed for 10 min at 37 °C in the presence of various concentration of *B. lanceolatus* or *B. atrox* venoms (1–1000 ng/mL) and placed on 96-well plates. Then, 33 µL of pre-warmed standard coagulation control plasma was added to each well. To assess the efficacy of the antivenom, Bothrofav^®^ was used at a concentration of 21 mg/mL in the presence of the highest concentration of venoms used herein. The turbidity caused by fibrin formation was continued for 20 min at 405 nm, using a microplate reader spectrophotometer (AMR-100, Allsheng, Hangzhou, China). Then, 3 independent experiments of 8 replicates for each tested condition were performed.

To determine the fibrinogen clotting activity, 100 µL of a solution containing human purified fibrinogen (6-FIB-5, Cryopep, Montpellier, France) at 1.1 mg/mL was added to pre-warmed HEPES-saline buffer (10 mM HEPES, 150 mM NaCl, pH = 7.4), CaCl_2_ (2.5 mM) containing 1000 ng/mL or 10^5^ ng/mL of *B. lanceolatus* or 1000 ng/mL of *B. atrox* venoms. The fibrin formation was assessed as described above. One experiment was performed in triplicate for each set of conditions.

### 5.5. Fibrinogen Cleavage Assay

The fibrinogenolytic activity of *B. atrox and B. lanceolatus* was determined, as described below: human fibrinogen (30 µg) was incubated in 10 mM of HEPES-saline buffer containing 150 mM NaCl, pH = 7.4 and 2.5 mM CaCl_2_ at 37 °C for 10, 20, 30, and 60 min with 1000 ng/mL of *B. atrox* venom and 1000 ng/mL or 10^5^ ng/mL of *B. lanceolatus* venom. After the incubation period, the reaction was stopped by the addition of denaturing buffer containing dithiothreitol (10 mM) and heating the samples at 100 °C for 5 min. All samples were subjected to 12% SDS-PAGE under reducing conditions for the analysis of fibrinogen degradation [41]. The molecular mass standard (Precision Plus Protein™ Kaleidoscope™ Prestained Protein Standards #1610375 and Precision Plus Protein™ All Blue Prestained Protein Standards #1610373, BioRad) and a 10 × 10 Mini-PROTEAN Tetra Cell System (BioRad, Grabels, France) electrophoresis system with a PowerPac™ HC (BioRad, Grabels, France) power supply was used. The protein fragments were stained using Coomassie Brilliant Blue R250 and visualized using an Amersham Imager 600 (GE Healthcare Life, Versailles, France). Two independent experiments were performed.

### 5.6. Evaluation of Coagulation Factors Activation

The kinetics of the activation of coagulation factors (prothrombin, FX) and kallikrein by *B. lanceolatus* and *B. atrox* venoms (1–1000 ng/mL) against a control were assessed using specific chromogenic substrates, p-nitroaniline (pNA) peptides (pNAPEP): pNAPEP 0216 (Tos-Gly-Pro-Arg-pNA), pNAPEP 1032 (Suc-Ile-Glu(γPip)-Gly-Arg-pNA), and pNAPEP 1266 (H-D-Val-Leu-Arg-pNA), respectively (Cryopep, Montpellier, France), and then recorded every 1 min for 30 min at 405 nm, using a microplate reader spectrophotometer (AMR-100, Allsheng, Hangzhou, China). The use of a synthetic chromogenic substrate for the measurement of specific coagulation factor activity needs to consider both (i) the presence of enzymes in the crude venom that can cleave a specific chromogenic substrate and (ii) the effects of venom added to plasma that can cleave coagulation factor zymogens, which then lead to their activation, thereby leading to cleavage of the specific chromogenic substrate. For example, the specific pNAPEP 0216 peptide can be cleaved due to venom thrombin-like activity, but when the venom is added to plasma, it may or may not be able to activate the coagulation cascade leading to thrombin formation from prothrombin. Hence, pNA release was assessed in plasmatic conditions using a normal plasma control diluted at 1/5 in Tris-BSA buffer containing 2.5 mM of CaCl_2_, 0.0025 mM of phospholipids, and 0.2 mM of pNAPEP, or assessed under the same conditions without plasma.

In order to avoid the interference of fibrin formation with OD measurement at 405 nm, Pepbloc FG (Cryopep, Montpellier, France) was added at a final concentration of 0.125 U/mL in all conditions. Pepbloc FG binds to fibrinogen to inhibit the polymerization of the fibrin network. *B. lanceolatus* and *B. atrox* venoms were used at final concentrations ranging from 1 to 1000 ng/mL. To assess the specific activation of the coagulation factor by the venom, the difference in optical density (OD), quantifying pNA release between condition with and without plasma, was calculated (*n* = 4–8 for each set of conditions).

The ability of the venoms to directly activate prothrombin and FX was also studied using the same experiment. In this case, the plasma was replaced by 200 µg/mL of purified human prothrombin (Cryopep, Montpellier, France) or 10 µg/mL of purified human FX (Cryopep, Montpellier, France), and pNAPEP 0216 and 1032 were used, respectively. The *B. lanceolatus* or *B. atrox* venoms were used at 1000 ng/mL. Similarly, 1000 ng/mL of the prothrombin-activator venom protease ecarin and 1 UI /mL of the coagulation FX activating enzyme from Russell’s viper venom (RVV-X) were used as positive controls (*n* = 4 for each set of conditions).

### 5.7. Thrombus Formation Analysis in Flow Conditions

Thrombus formation was assessed using the T-TAS (Fujimori Kogyo Co., Tokyo, Japan). This flow chamber system and its dedicated microchips (AR-Chip) reproduce the thrombus formation reaction in ex vivo blood samples [22]. The AR-Chip is pre-coated with type-I collagen and tissue thromboplastin. This microchip is designed to assess primary and secondary hemostasis under flow conditions. A whole citrated blood sample (480 µL) was mixed with 20 µL of calcium corn trypsin inhibitor (CaCTI) immediately before transferring 450 µL of the mix into the AR-Chip. CaCTI was used to recalcify the citrated blood sample and inhibit the signaling pathway by binding to factor XII. For the AR-Chip, thrombus formation occurs under 600/s shear stress, occluding the microchip, while the inner pressure increases (Figure A2). Thrombus formation within the flow chamber increases flow resistance, causing the pressure to increase. OST (occlusion start time) represents the lag time for the flow pressure to reach 10 kPa, due to the partial occlusion of the capillary. Thrombus formation growth rates (T_10_–T_60_) refer to the lag time for the flow pressure to reach 60 kPa from 10 kPa of pressure [22]. Occlusion time (OT) refers to the lag time for the flow pressure to reach 60 kPa from the baseline pressure [22]. The concentrations used for the *B. lanceolatus* experiments (with or without Bothrofav^®^) ranged from 50 to 1000 ng/mL. Concentrations of 500 ng/mL and 1000 ng/mL were used in the *B. atrox* venom experiments.

### 5.8. Statistical Analysis

The data reported herein represent the mean ± standard deviation (SD). All statistical analyses were performed with GraphPad PRISM 9.5 (Graphpad Prism Inc., San Diego, CA, USA). The samples were compared using a one-way analysis of variance (ANOVA). A value of *p* < 0.05 indicated statistical significance. When a significant difference was found, we identified specific differences between the groups using Bonferroni post hoc analysis.

## Data Availability

Data are available from the corresponding author upon reasonable request.

## Appendix A

**Table A1:** Summary table with the conclusions from each experiment performed.

		*B. lanceolatus*	*B. atrox*
Procoagulant effect			
	Static conditions	From 250 ng/mL	From at least 1 ng/mL
	Flow condition (clot firmness)	+/−	+
	Neutralization	++	+/−
Clotting factor activation			
	Prothrombin	+	++
	Factor X	−	++
	Prekallikrein	+	++
	Fibrino(geno)lytic activity	+/−	++
Amidolytic activity			
	Thrombin-like	++	−
	FXa-like	−	−
	Kallikrein-like	++	−

+ moderate change; ++ major change, +/− non-significant change, and − no change compared with controls.
